# Impact of the SARS-COV-2 outbreak on epidemiology and management of major traumain France: a registry-based study (the COVITRAUMA study)

**DOI:** 10.1186/s13049-021-00864-8

**Published:** 2021-03-22

**Authors:** Jean-Denis Moyer, Arthur James, Clément Gakuba, Mathieu Boutonnet, Emeline Angles, Emmanuel Rozenberg, Jean Bardon, Thomas Clavier, Vincent Legros, Marie Werner, Quentin Mathais, Véronique Ramonda, Pierre Le Minh, Yann Berthelot, Clélia Colas, Julien Pottecher, Tobias Gauss, and the Traumabase Group

**Affiliations:** 1 Department of Anesthesiology and Critical Care, Beaujon Hospital, DMU Parabol, AP-HP.Nord, 100 boulevard du General Leclerc, F92110 Clichy, France; 2 Department of Anaesthesiology and critical care, Pitié-Salpêtrière Hospital, Sorbonne University, GRC 29, AP-HP, DMU DREAM, Paris, France; 3 Department of Anesthesiology and Critical Care Medicine, Caen University Hospital, Avenue de la cote de Nacre, Caen, France; 4 Intensive Care Unit, Percy Military Teaching Hospital. 101 avenue Henri Barbusse 92140, Clamart, Val de Grace Academy, place Alphonse Laveran, 75005 Paris, France; 5 Department of Anesthesiology and Critical Care, Bordeaux University Hospital, Pellegrin, Bordeaux, France; 6 Department of Anesthesiology and Critical Care, Hôpital Européen Georges Pompidou, Paris, France; 7 Department of Anesthesiology and Critical Care, Hôpital Henri Mondor, Créteil, France; 8 Department of Anesthesiology and Critical Care, Rouen University Hospital, 1 rue de Germont, 76000 Rouen, France; 9 Department of Anesthesiology and Critical Care, Hopital Maison Blanche – CHU de Reims, Reims, France; 10 Department of Anesthesiology and Critical Care, APH-HP, Bicêtre Hôpitaux Universitaires Paris-Sud, Université Paris Saclay, Le Kremlin Bicêtre, France; 11 Department of Anesthesiology and Critical Care, Military Teaching Hospital, Sainte-Anne, Toulon, France; 12 Department of Anesthesiology and Critical Care, University Toulouse 3-Paul-Sabatier, University Hospital of Toulouse, Hôpital Pierre-Paul Riquet, CHU Toulouse-Purpan, 31059 Toulouse, France; 13 Capgemini Invent, Insight Driven Enterprise, focused on Data & Artificial Intelligence services, Issy-les-Moulineaux, France; 14 Hôpitaux Universitaires de Strasbourg, Pôle d’Anesthésie-Réanimation & Médecine Péri-Opératoire, Service d’Anesthésie-Réanimation & Médecine Péri-Opératoire Hôpital de Hautepierre - Université de Strasbourg, Faculté de Médecine, Fédération de Médecine Translationnelle de Strasbourg (FMTS), UR3072, Strasbourg, France

**Keywords:** COVID-19, Trauma, France, Traumacenter

## Abstract

**Background:**

Emerging evidence suggests that the reallocation of health care resources during the COVID-19 pandemic negatively impacts health care system. This study describes the epidemiology and the outcome of major trauma patients admitted to centers in France during the first wave of the COVID-19 outbreak.

**Methods:**

This retrospective observational study included all consecutive trauma patients aged 15 years and older admitted into 15 centers contributing to the TraumaBase® registry during the first wave of the SARS-CoV-2 pandemic in France. This COVID-19 trauma cohort was compared to historical cohorts (2017–2019).

**Results:**

Over a 4 years-study period, 5762 patients were admitted between the first week of February and mid-June. This cohort was split between patients admitted during the first 2020 pandemic wave in France (pandemic period, 1314 patients) and those admitted during the corresponding period in the three previous years (2017–2019, 4448 patients). Trauma patient demographics changed substantially during the pandemic especially during the lockdown period, with an observed reduction in both the absolute numbers and proportion exposed to road traffic accidents and subsequently admitted to traumacenters (348 annually 2017–2019 [55.4% of trauma admissions] vs 143 [36.8%] in 2020 *p* < 0.005). The in-hospital observed mortality and predicted mortality during the pandemic period were not different compared to the non-pandemic years.

**Conclusions:**

During this first wave of COVID-19 in France, and more specifically during lockdown there was a significant reduction of patients admitted to designated trauma centers. Despite the reallocation and reorganization of medical resources this reduction prevented the saturation of the trauma rescue chain and has allowed maintaining a high quality of care for trauma patients.

**Supplementary Information:**

The online version contains supplementary material available at 10.1186/s13049-021-00864-8.

## Background

In December 2019 Wuhan, China, experienced an outbreak of coronavirus disease 2019 (COVID-19), caused by severe acute respiratory syndrome coronavirus 2 (SARS-CoV-2) [1, 2]. This outbreak was declared a pandemic by World Health Organization, and has spread to the entire World. France was not spared and the number of COVID-19 cases has grown since the end of January 2020 and recently reached his climax in April 2020. In order to stem the spread of SARS CoV-2, the French government imposed a first national lockdown in France from March 13, 2020 for duration of 55 days.

Despite extensive public health interventions, a large number of patients were admitted to French intensive care units, mainly with acute respiratory distress syndrome (ARDS). As a consequence of the massive and constant influx of patients SARS-CoV-2 pandemic had a major impact on health care system. The COVID-19 led to a massive increase in the demand for acute care beds and challenged existing surge capacity. This required a fast and thorough restructure of the health care system [3]. The national plan for healthcare institutions (named “Plan Blanc”) was deployed in order to obtain a long-lasting increase of the total number of available hospital beds. A central element consisted in the postponement of non-urgent medical consultations and interventions allowing the reallocation of manpower and resources to COVID-19 patients [4].

The impact on the French trauma system was expected to be important, considering that all three key actors namely prehospital emergency services, intensive care units and anesthesiology services, were highly involved in the management of COVID-19 patients. ICU capacity usually dedicated to trauma care was reassigned to COVID-19 and other critical care admissions patients. Anesthesiologists and nursing colleagues from operating theatres were reallocated to existing or newly created critical care units. This reorganization exposed to a potential shortage of essential resources, destabilization of the trauma system and inappropriate or even deleterious delays in definitive care.

Potential mitigation was offered as a consequence of the national lockdown reducing movement and road traffic accident but in turn potentially offset by increasing numbers of assaults or fall from height secondary to suicide attempts [5, 6].

Altogether, these indirect effects of the COVID-19 pandemic carried the risk of detrimental effects on trauma networks with an increase of trauma related mortality. Emerging evidence suggests that the effect of the SARS-CoV-2 pandemic on overall mortality will be complex and beyond the direct consequences of the disease [7–9].

For these reasons, this study set out to explore the impact of the COVID-19 pandemic and the subsequent reorganization of the national health care system on the epidemiology, management and mortality of trauma patient admitted to trauma centers across France in the first wave of 2020. The results may identify areas of improvement for future pandemics waves and inform public health policy decisions.

## Methods

### Study design

We conducted a multicentric cohort-based observational study including data from 2017 to 2020. During each of these 4 years, all consecutive trauma admitted into 15 regional trauma centers participating in the Traumabase registry (Additional file 1) from the first week of February to the end of the second week of June were included. This study is reported according to the STROBE guidelines for observational studies [10] (Additional file 2).

### Data source

The Traumabase registry prospectively collects socio-demographic, clinical, biological, therapeutic, and in-hospital evolution data for all severely injured patients admitted to a participating center and suspected of severe trauma. For each patient, data collection ranges from the prehospital scene to hospital discharge. Every participating center admits all consecutive severe trauma occurring in their respective geographical area allowing for a cohort-based overview of severe trauma care in each given area. Severe trauma is defined as a situation suggesting life threatening or changing injuries (Additional file 3) [11].

### Data analysis

Patients were stratified according to their year of admission, allocating those admitted the years 2017, 2018 and 2019 in one group (non-pandemic period) and those admitted in 2020 into another group (pandemic period).

To explore the effect of the lockdown, 3 periods were defined for each of these years:

- the *pre-lockdown*: week 6 to week 12 (respectively lasting from the beginning of the first week of February to the end of the second week of March (2017–2019) and from the February 3rd to March 16th for 2020),
- the *lockdown*: week 12 to week 20 (respectively lasting from the beginning of the third week of March to the end of the end of the second week of May (2017–2019) and from March 17th to May 10th for 2020),
- and the *post-lockdown*: week 20 to week 24 (respectively lasting from the beginning of the third week of May to the end of the second week of June (2017–2019) and from the May 11th to June 15th for 2020).

For each year, these 3 periods were divided in 18 consecutive weeks. These consecutive weeks were matched by calendar weeks allowing for comparisons across similar segments of time across the year 2020 and previous years [eg: Weeks 16 of the previous years can be compared to Week 16 of the year 2020].

### Outcomes

For each of the 3 periods, the number of patients admitted in the participating centers is reported. For each patient, age, gender, injury mechanism, injury severity (Injury Severity Score (ISS)) [12], new Simplified Acute Physiology Score (SAPS2) [13], haemorrhagic shock (transfusion of more than four blood products within 6 h) [14] and traumatic brain injury (TBI) (intracranial bleeding on CT scan), trauma management (pre-hospital and intra-hospital time, surgery during the first 24 h, duration of mechanical ventilation length, length of stay and decision of withdrawal of care decisions) and mortality are reported. The Trauma ISS (TRISS) is used to compare the observed mortality (in-hospital mortality) to the predicted mortality [15].

### Statistical analysis

Variables with an overall proportion of missing data > 10% between 2020 and previous years were not included. Missing data were not imputed and are reported for each variable in Additional file 4. All statistics were computed using Python and the Traumabase®registry; no patients were excluded. Continuous variables are described by mean and standard deviation, whereas categorical variables are described in number (percentage). Each variable result over the previous years (2017, 2018 and 2019) were summarized using its mean. Significance tests were computed at a level of confidence of 95% using: a non-parametric test of Mann-Whitney for continuous variables, and a Chi-2 test for discrete and categorical ones. The comparisons were made between 2020 and the previous years (2017–2019) on each period (pre-lockdown, lockdown and post lockdown). In order to adjust for the repeated application of statistical tests, the level of significance was set at *p* < 0.01.

## Results

Over the 4 year-period, 5762 patients were admitted between the first week of February and the end of the second week of June. This cohort was split between patients admitted during this time frame in 2020 (1314 patients) and those admitted during the same time frame the 3 previous years (2017–2019, 4448 patients).

Tables 1 illustrate the baseline characteristics of both cohorts.

**Table 1:** Epidemiology of patients admitted at Traumacenter in 2020 and compared to previous years

	Period					
	Pre-lockdown (February 3rd – March 16th)		Lockdown (March 17th – May 10th)		Post-lockdown (May 11th – June 15th)	
Variable	Previous years	2020	Previous years	2020	Previous years	2020
Number of admitted patients	404	501	628	361	434	436
Age (Years)	41.5 (19.1)	42.7 (20.1)	41.5 (19.0)	43.2 (19.9)	39.6 (18.7)	39.6 (18.9)
Gender Female)	92 (22.8)	101 (20.2)	136 (21.7)	65 (18)	92 (21.2)*	65 (14.9)*
Penetrating trauma	58 (14.4)	63 (12.6)	68 (10.8)	53 (14.7)	50 (11.5)	52 (11.9)
Mechanism of injury distribution						
Road traffic accident	202 (50)	237 (47.3)	348 (55.4)*	133 (36.8)*	245 (56.5)	231 (53)
Fall from height	92 (22.7)	108 (21.5)	126 (20.1)*	128 (35.5)*	84 (19.4)	97 (22.2)
Aggression	74 (18.3)	84 (16.8)	90 (14.3)*	63 (17.4)*	65 (15)	69 (15.8)
Fall from standing	19 (4.7)	23 (4.6)	30 (4.8)*	23 (6.4)*	14 (3.2)	16 (3.7)
Other	15 (3.7)	16 (3.2)	25 (4)*	11 (3)*	18 (4.1)	19 (4.4)
ISS	15.6 (12.6)	15.0 (12.5)	15.8 (13.4)	16.4 (12.6)	15.0 (11.9)	16.2 (12.3)
SAPS 2	27.1 (19.4)	26.6 (19.3)	27.8 (20.0)	27.9 (19.1)	26.3 (19.2)	27.1 (18.8)
Hemorrhagic shock	24 (5.9)	25 (5)	46 (7.3)	20 (5.5)	27 (6.2)	22 (5)
Traumatic brain injury	109 (27)	107 (21.4)	152 (24.2)	87 (24.1)	105 (24.2)	108 (24.8)

* Difference between 2020 and Previous years significant with *p* value a ≤ 0.01

Data are N (%), mean (Standard Deviation);

*ISS* Injury Severity Score, *SAPS 2* Simplified Acute Physiology Score

### Pre-lockdown period

#### Patients characteristics

**Fig. 1:**
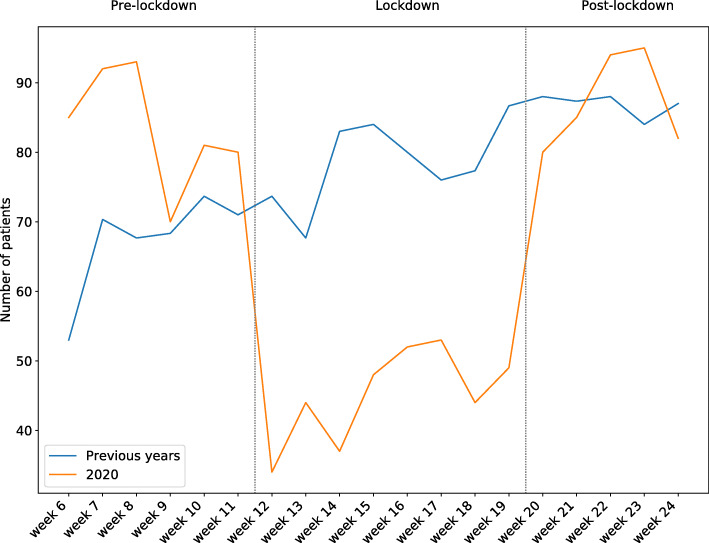
Weekly number of patients admitted in Traumacenter in 2020 and compared with previous years (average of 2017–2019)

Admissions remained stable during *pre-lockdown* (404 admissions during the previous years vs 501 in 2020) (Fig. 1). There was no difference in patients ‘characteristics and severity (Table 1).

#### Trauma management

Helicopter transportation (49 [12.1%]vs 39 [7.8%] *p* = 0.0007) and the rate of pre-hospital orotracheal intubation (85 [21%]vs 82 [16.4%] *p* = 0.002) decreased without any change in total pre-hospital time (81.9 min [71.4] vs 79.6 min [48.2] *p* = 0.866). Secondary admission from non-trauma center hospitals in previous years was equivalent to 2020 (72 [17.8%] vs 106 [21.2%] *p* = 0.035). The proportion of surgery performed within the first 24 h was comparable in both periods (193 [47.8%] vs 224 [44.7%] *p* = 0.029).

### Lockdown period

#### Patients characteristics

**Fig. 2:**
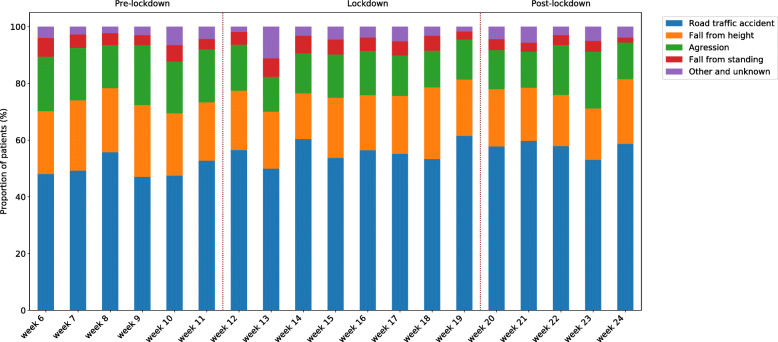
Weekly distribution of injury mechanism admitted at Traumacenter during the previous years (average of 2017 to 2019)

**Fig. 3:**
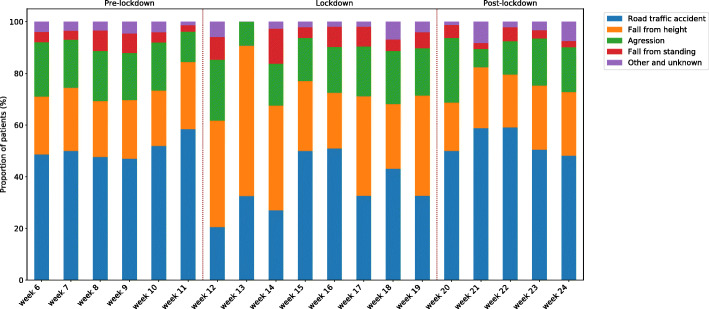
Weekly distribution of injury mechanism admitted at Traumacenter in 2020

The comparison highlights a significant reduction of overall admissions during the 2020 period (628 admissions during the previous years vs 361 in 2020) (Fig. 1) (Table.1). The absolute number and proportion of road traffic accidents decreased during the *lockdown* (348 [55.4%] vs 143 [36.8%] in 2020 *p* < 0.005) while other mechanisms remained stable in absolute numbers (fall from height, assault, fall from standing) (Figs. 2 and 3) (Table 1). Injury severity evaluated by ISS (15.8 [13.4] vs 16.4 [12.6] *p* = 0.126), SAPS 2 (27.9 [19.1] vs 27.8 [20.0], *p* = 0. 415) and incidence of traumatic brain injury (152 [24.2%] vs 87 [24.1%] *p* = 0.704) and hemorrhagic shock (46 [7.3%]vs 20 [5.5%] *p* = 0.350) were not different from previous years.

#### Trauma management

Prehospital care and hospital care were similar between 2020 and previous years (Table 2). The only difference was an increase in the time in the trauma bay in 2020 (29 min [28.7] vs 33.5 min [46] *p* = 0.006). The proportion of patients requiring surgical intervention in the first 24 h was identical (311 [49.5] vs 181 [50.1%] *p* = 0.418). Length of stay in the intensive care unit (ICU) (5.8 days [14.8] vs 6.8 days [13.8] *p* = 0.155) and duration of mechanical ventilation (7.5 [11.7] days vs 9.6 [15.9] days *p* = 0.154), were comparable to the previous years.

**Table 2:** Prehospital and hospital management patients admitted at Traumacenter in 2020 and compared to the previous years

	Period					
	Pre-Lockdown (February 3rd – March 16th)		Lockdown (March 17th – May 10th)		Post-Lockdown (May 11th – June 15th)	
Variable	Previous years	2020	Previous years	2020	Previous years	2020
Observed Mortality (in hospital)	35 (8.6)	33 (6.6)	62 (9.9)	21 (5.8)	34 (7.8)	38 (8.7)
Predicted mortality (TRISS) (%)	10.8 (22.7)	10.3 (22.1)	11.0 (23.1)	11.7 (23.6)	10.4 (21.6)	9.4 (21.2)
Variations of the difference between the predicted mortality (TRISS) and observed mortality	0.1 (0.2)	0.1 (0.2)	0.1 (0.2)	0.1 (0.2)	0.1 (0.2)	0.1 (0.2)
Transportation to hospital (helicopter)	49 (12.1)*	39 (7.8)*	84 (13.4)	38 (10.5)	67 (15.4)	67 (15.4)
Pre-hospital orotracheal intubation	85 (21)*	82 (16.4)*	131 (20.9)	70 (19.4)	98 (22.6)	87 (20)
Pre-hospital time (min)	81.9 (71.4)	79.6 (48.2)	80.4 (82.4)	80.0 (45.9)	76.6 (50.4)	81.2 (47.8)
Intra-hospital time (min)	28.4 (26.5)	31.31 (37.0)	29.0 (28.7)*	33.5 (46.0)*	27.2 (26.3)	27.0 (18.0)
Surgery in the first 24 h (%)	193 (47.8)	224 (44.7)	311 (49.5)	181 (50.1)	212 (48.8)	217 (49.8)
Immediate surgical or arteriography intervention	14 (3.5)	6 (1.2)	21 (3.3)	8 (2.2)	15 (3.5)	13 (3)
Length of mechanical ventilation (days)	8.8 (14.3)	7.5 (11.2)	7.5 (11.7)	9.6 (15.9)	8.2 (16.3)	6.5 (9.1)
Length of stay (days)	6.4 (13.5)	5.1 (8.8)	5.8 (14.8)	6.8 (13.8)	6.6 (20.3)	5.2 (8.7)
Admission from non trauma center hospital	72 (17.8)	106 (21.2)	94 (15)	55 (15.2)	65 (15)	76 (17.4)
Patient with decisions of withdrawal of care	23 (5.7)	14 (2.8)	34 (5.4)	18 (5)	19 (4.4)	25 (5.7)

* Difference between 2020 and Previous years significant with *p* value ≤0.01

Data are N (%), mean (SD)

*TRISS* Trauma Related Injury Severity Score

### Post lockdown period

#### Patients characteristics

In *post-lockdown*, more women were affected by major trauma in 2020 (92 [21.2%] vs 65 [14.9%] *p* = 0.004) while the number of road traffic accidents (245 [56.5%] vs 231 [53%] *p* = 0.202) was equivalent to the previous years. There was no difference in severity evaluated by ISS (15 [11.9] vs 16.2 [12.3] *p* = 0.04) and SAPS 2 scores (27.1 [18.8] vs 26.3 [19.2] *p* = 0.146) (Table 1).

#### Trauma management

Prehospital care and hospital care did not differ between pandemic and non-pandemic periods (Table 2).

### Mortality

**Fig. 4:**
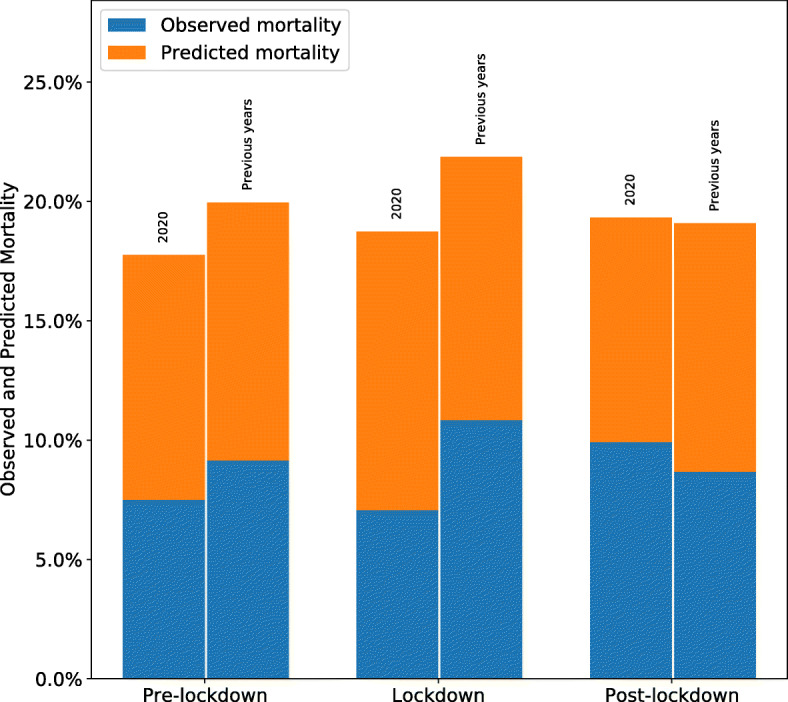
Observed mortality and expected mortality during pre-lockdown, lockdown and post-lockdown in 2020 and compared to the previous years (average of 2017–2019)

The in-hospital observed mortality between previous years and 2020 remained stable throughout all three phases (Table 2) (Fig. 4). No significant difference was noted between observed and predicted mortality rates among any of the study periods (Table 2) (Fig. 4).

## Discussion

### Overview

To our knowledge this is the largest study on a national scale to explore the impact of the COVID-19 pandemic and the associated reallocation of health care resources on the care of major trauma patients. The results demonstrate a significant impact on the epidemiology of trauma in France during lockdown. The national lockdown reduced the overall trauma incidence in particular the frequency of road traffic accidents. The reallocation of critical care resources did not substantially alter the capacity of the health care system to provide high standards of care for the severely injured.

Several factors may explain this observation. During this COVID-192020 spring wave, a substantial volume of critical care capacity was reallocated to absorb COVID-19 patients with the risk to reduce capacity available for major trauma [4]. In France, the vast majority of designated trauma centers are also the regional referral or tertiary care centers, suggesting a potential competition between patient cohorts for critical care resources. However as demonstrated by Lefrant et al. this potential effect was partially compensated by the surge in intensive care beds [16]. Furthermore, the results document the national lockdown generated a sufficient reduction in overall trauma incidence of 43% compared to previous years. This decrease obviously prevented the overflow of trauma centers.

The injury patterns and patient characteristics in the period immediately before, during and after lockdown are very similar to the corresponding period in the previous years. The reduction in road traffic accidents was not accompanied by a surge in assault or suicide; except for road traffic accidents, all trauma mechanisms conveyed a similar number of injured victims. Trauma centers did not restrict their admission policy, as indicated by the similar median ISS across the study period, injury patterns were identical.

In terms of the quality of care, prehospital transport time did not seem different from the previous years [17]. The rate of helicopter transportation and prehospital intubations decreased slightly during the pre-lockdown period. This observation could reflect a transient adaptation during the pre-lockdown period (representing the impact of SARS-CoV-2) or a just a natural variation.

Time in the trauma bay increased slightly during lockdown, but the effect was probably minimal on patient outcome. The proportion of patients operated within the first 24 h remained identical suggesting timely access to complex trauma surgery was not impeded. The observed mortality and predicted mortality remained stable and comparable to previous years during this spring surge suggesting that the prehospital to intrahospital 24-h rescue chain and subsequent critical care capacity remained intact for major trauma. The trauma systems in the areas assessed in this study appeared resilient enough to absorb the shock of the 2020 COVID-19 spring surge and provide adequate and appropriate care equivalent to previous years.

These reassuring results will require re-evaluation in the case of any renewed surge of the pandemic. In fact, this first wave was characterized by a complete and imposed stop of all elective surgical and medical procedures to free up crucial resources. A new surge may not lead again to a complete cessation of interventional activity in order to provide the complete spectrum of medical care in particular for oncological cases. As stated above, the study by Lefrant et al. reported 4806 newly created ICU beds (+ 95% increase) in France [16]. Without these adjustments, the strain on the trauma care networks could have been far higher and could have altered the level of care provided.

### Limitations

The authors acknowledge some limitations inherent to the exceptional context and observational retrospective nature of the study. The results are based on data collected in fifteen trauma centers, with six centers contributing from the Paris region alone. This selection of a mix from urban and rural areas may not be representative of the entire French territory. However, the centers included are the level-1 referral centers in two of the most highly affected pandemic areas during the 2020 spring wave (Ile de France, Grand Est). The increase and intense clinical workload during the study period may have affected the capacity of clinicians and research assistants to collect data. Third, the database does not provide any data on the long-term and functional outcome of patients after TBI (such as the Glasgow Outcome Scale Extended (GOS-E) for TBI or quality of life). Consequently, we were unable to evaluate any significant outcomes other than in-hospital mortality. Moreover, the proportion of trauma patients infected with COVID-19 is unknown and the impact on their care and outcome cannot be evaluated. In addition, this study only considers patients who were admitted alive at the hospital. It is impossible to assess whether the number of severely traumatized patients who died in the pre-hospital setting has not increased [18].

## Conclusions

Our study provides an insight into the epidemiology and management of trauma patients during the 2020 COVID-19 pandemic in France for the most affected areas. During this period and more specifically during lockdown, the study demonstrated a 50% reduction in road traffic accidents with no increase in alternative injury mechanisms, such as assault or suicide. The in-hospital observed and predicted mortality and a number of crucial process indicators remained stable compared to previous years suggesting a sufficient resilience of the trauma networks assessed to absorb the spring 2020 pandemic hit. This study suggests that the care for major trauma patients was not substantially impacted by the SARS-CoV-2,2020 first phase in France.

## Supplementary Information

**Additional file 1: **Participating centers.

**Additional file 2: ** STROBE checklist.

**Additional file 3.** Vittel Criteria.

**Additional file 4.** Missing data _proportion by variables (%)_.

## Data Availability

The data that support the findings of this study are available from the Traumabase group but restrictions apply to the availability of these data, which were used under license for the current study, and so are not publicly available. Data are however available from the authors upon reasonable request and with permission of the Traumabase group.

## Abbreviations

COVID-19: Coronavirus disease 2019
SARS-CoV-2: Severe acute respiratory syndrome coronavirus 2
ARDS: Acute respiratory distress syndrome
ISS: Injury severity score
SAPS2: Simplified Acute Physiology Score
TBI: Traumatic brain injury
ICU: Intensive care unit
