# Supplementary material for: Impact of the SARS-COV-2 outbreak on epidemiology and management of major traumain France: a registry-based study (the COVITRAUMA study)
Source: Scand J Trauma Resusc Emerg Med. 2021 Mar 22;29:51. doi: 10.1186/s13049-021-00864-8 (PMC7983347; doi:10.1186/s13049-021-00864-8)
Supplement: Supplementary file 1 — Additional file 1: Participating centers. [file 13049_2021_864_MOESM1_ESM.docx]

## **Additional files**

## Supplementary material 1: 15 Participating centers

1.Department of Anesthesiology and Critical Care, AP-HP Beaujon,

2. Department of Anesthesiology and Critical Care, AP-HP Bicétre

3.Department of Anesthesiology and Critical Care, AP-HP HEGP

4.Department of Anesthesiology and Critical Care, AP-HP Mondor

5.Department of Anesthesiology and Critical Care, AP-HP Pitié-Salpêtrière

6. Department of Anesthesiology and Critical Care Metz Thionville

7.Department of Anesthesiology and Critical Care, CHU Nancy

8.Department of Anesthesiology and Critical Care, CHU Toulouse Purpan

9.Department of Anesthesiology and Critical Care, CHU Toulouse Rangeuil

10.Department of Anesthesiology and Critical Care, CHU Caen

11.Department of Anesthesiology and Critical Care, CHU Reims

12.Department of Anesthesiology and Critical Care, CHU Rouen

13.Department of Anesthesiology and Critical Care, HIA, Percy

14.Department of Anesthesiology and Critical Care, HIA Saint-Anne

15.Department of Anesthesiology and Critical Care, Hopital Civil de Colmar
