# Supplementary material for: Impact of the SARS-COV-2 outbreak on epidemiology and management of major traumain France: a registry-based study (the COVITRAUMA study)
Source: Scand J Trauma Resusc Emerg Med. 2021 Mar 22;29:51. doi: 10.1186/s13049-021-00864-8 (PMC7983347; doi:10.1186/s13049-021-00864-8)
Supplement: Supplementary file 3 — Additional file 3. Vittel Criteria. [file 13049_2021_864_MOESM3_ESM.docx]

*Additional file 3: Vittel Criteria*

**Step 1 (Physiological signs)**

GCS < 13

SAP < 90 mmHg

SpO2 < 90%

**Step 2 (Global assessment of speed and mechanism)**

Ejection from vehicle

Death in same passenger compartment

Fail > 6 m

Victim thrown or projected

Global assessment of speed and potential injuries:

Vehicle deformation, estimated vehicle speed no helmet, no seat belt

Blast

**Step 3 (Anatomical injuries)**

Penetrating trauma of head, neck, thorax, abdomen, arms or legs

Flail chest

Severe burn

Pelvic fracture

Suspicion of spinal cord injury

Amputation at or above wrist or ankle level

Acute limb ischemia

**Step 4 (resuscitation)**

Mechanical ventilation

Intravascular filling > 1000 ml

Vasopressor
