# Supplementary material for: Impact of the SARS-COV-2 outbreak on epidemiology and management of major traumain France: a registry-based study (the COVITRAUMA study)
Source: Scand J Trauma Resusc Emerg Med. 2021 Mar 22;29:51. doi: 10.1186/s13049-021-00864-8 (PMC7983347; doi:10.1186/s13049-021-00864-8)
Supplement: Supplementary file 4 — Additional file 4. Missing data proportion by variables (%). [file 13049_2021_864_MOESM4_ESM.docx]

## Additional file 4: Missing data proportion by variables (%)

For table 1

| Variable | Year | Period  Pre-Lockdown | Lockdown | Post-Lockdown | Whole year |
| --- | --- | --- | --- | --- | --- |
| Injury mechanism | 2020 | 6.38 | 0.83 | 0.92 | 3.04 |
|  | Previous years | 0.58 | 1.38 | 1.92 | 1.32 |
| Age | 2020 | 0 | 0 | 0 | 0 |
|  | Previous years | 0 | 0 | 0 | 0 |
| Sex | 2020 | 6.58 | 1.11 | 1.15 | 3.27 |
|  | Previous years | 0.66 | 1.43 | 1.99 | 1.38 |
| ISS | 2020 | 9.09 | 5.54 | 2.98 | 6.09 |
|  | Previous years | 5.20 | 4.14 | 3.61 | 4.27 |
| TRISS | 2020 | 32.49 | 25.21 | 20.64 | 26.56 |
|  | Previous years | 18.65 | 18.57 | 19.11 | 18.75 |
| IGS | 2020 | 6.58 | 6.37 | 3.21 | 5.40 |
|  | Previous years | 1.07 | 2.12 | 2.07 | 1.82 |
| Hemorrhagic shock | 2020 | 7.35 | 6.37 | 2.52 | 5.48 |
|  | Previous years | 1.32 | 2.07 | 2.61 | 2.02 |
| Traumatic Brain Injury | 2020 | 7.54 | 6.93 | 2.75 | 5.78 |
|  | Previous years | 1.76 | 2.02 | 2.76 | 2.16 |

| Variable | Year | Period  Pre-Lockdown | Lockdown | Post-Lockdown | Whole year |
| --- | --- | --- | --- | --- | --- |
| Mortality | 2020 | 12.19 | 17.73 | 12.16 | 13.70 |
|  | Previous years | 6.11 | 8.44 | 9.82 | 8.2 |
| Transportation to hospital | 2020 | 9.28 | 3.60 | 3.21 | 5.71 |
|  | Previous years | 1.4 | 2.33 | 2.69 | 2.18 |
| Pre-hospital orotracheal intubation | 2020 | 10.83 | 3.60 | 2.98 | 6.24 |
|  | Previous years | 2.06 | 2.71 | 3.38 | 2.73 |
| Pre-hospital time | 2020 | 11.41 | 5.26 | 4.59 | 7.46 |
|  | Previous years | 4.21 | 3.5 | 4.37 | 3.95 |
| Intra-hospital time | 2020 | 31.91 | 21.88 | 21.79 | 25.80 |
|  | Previous years | 21.95 | 20.05 | 21.26 | 20.93 |
| Surgical interventions in the first 24h | 2020 | 8.12 | 4.43 | 3.21 | 5.48 |
|  | Previous years | 2.48 | 3.08 | 3.68 | 3.09 |
| Surgical or arteriography intervention | 2020 | 28.05 | 18.56 | 19.27 | 22.53 |
|  | Previous years | 20.13 | 17.51 | 17.88 | 18.34 |
| Mechanical ventilation duration | 2020 | 16.97 | 8.03 | 8.72 | 9.39 |
|  | Previous years | 7.92 | 9.18 | 11.05 | 12.33 |
| Hospitalization duration | 2020 | 8.70 | 14.68 | 14.68 | 12.33 |
|  | Previous years | 9.74 | 11.72 | 12.12 | 11.29 |
| Secondary admission | 2020 | 6.77 | 1.11 | 1.61 | 3.50 |
|  | Previous years | 1.65 | 2.12 | 2.99 | 2.25 |
| Decisions to withdraw of care | 2020 | 12.77 | 16.62 | 12.16 | 13.62 |
|  | Previous years | 7.10 | 8.86 | 10.13 | 8.75 |

For Table 2
